# Prevalence of Rheumatic Heart Disease in First-Degree Relatives of Index-Cases: A Systematic Review and Meta-Analysis

**DOI:** 10.5334/gh.1417

**Published:** 2025-03-10

**Authors:** Antonio Mutarelli, Guilherme Paes Gonçalves Nogueira, Alexandre Negrao Pantaleao, Alleh Nogueira, Bruna Giavina-Bianchi, Isabella M. Gonzalez Fonseca, Bruno R. Nascimento, Walderez O. Dutra, Robert A. Levine, Maria C. P. Nunes, On behalf of PRIMA Network

**Affiliations:** 1 School of Medicine, Federal University of Minas Gerais, Brazil; 2 Cardiac Ultrasound Lab, Massachusetts General Hospital, Harvard Medical School, Boston, Massachusetts, US; 3 State University of Piauí, Teresina, Brazil; 4 Hospital Israelita Albert Einstein, São Paulo, SP, Brazil; 5 Universidade Federal de Minas Gerais –Serviço de Cardiologia e Cirurgia Cardíaca do Hospital das Clínicas, Belo Horizonte, MG, Brazil; 6 Serviço de Hemodinâmica, Hospital Madre Teresa, Belo Horizonte, MG, Brazil; 7 Faculty of Infectious and Tropical Diseases, London School of Hygiene and Tropical Medicine, London, UK; 8 Laboratório Biologia das Interações Celulares, Depto. Morfologia, Instituto de Ciências Biológicas, Belo Horizonte, MG, Brazil

**Keywords:** Rheumatic heart disease, first-degree relative, family members, meta-analysis

## Abstract

**Background:**

Rheumatic heart disease (RHD) is the leading cause of cardiac death in children, with over 300,000 annual fatalities. Immunological, genetic, and environmental factors contribute to an increased risk of RHD. It remains unclear whether first-degree relatives have a higher prevalence of RHD compared to the general population in the same region.

**Methods:**

We performed a systematic review and meta-analysis of echocardiographic screening studies reporting the prevalence of RHD in family members of individuals with RHD or acute rheumatic fever. PubMed, Embase, Cochrane, and Lilacs databases were searched. RHD was classified as per the 2012 World Heart Federation criteria. Random-effects models assessed definite RHD prevalence in study groups.

**Results:**

Four of the 1,160 studies were included, with 776 first-degree relatives screened. Two studies were from Africa, one from South America, and one from Oceania. In the first-degree relatives of index cases, the prevalence of RHD was 7% (95% confidence interval [CI] 3.7–13). The control group, individuals screened with no known RHD cases in their family, had a lower prevalence than first-degree relatives (risk ratio [RR] 0.44, 95% CI 0.26–0.75). There was no difference in the prevalence of RHD among siblings and parents of cases.

**Conclusion:**

There is an overall prevalence of non-relatives from the respective region, which suggests that genetic predisposition may play a role. In future studies of RHD, the systematic screening of first-degree relatives should be considered with a better control group—socioeconomic, region, age, and sex-matched.

## Introduction

Rheumatic heart disease (RHD) is the most prevalent acquired heart condition among children and adolescents globally (1). It results from acute rheumatic fever (ARF), which may manifest clinically or subclinically, ultimately leading to chronic and progressive valvular disease, stroke, heart failure, and arrhythmias (2). Worldwide, RHD affects over 40 million individuals and is responsible for more than 300,000 deaths annually (3).

Secondary prophylaxis following an episode of ARF significantly reduces the likelihood of developing and progressing RHD (4). A recent double-blind controlled trial (5) indicated that regular intramuscular penicillin use notably decreases the risk of latent RHD progression, defined as asymptomatic valvular involvement detected by echocardiography. According to a global meta-analysis, the prevalence of latent RHD may be as high as 21 per 1000 and some subgroups, including rural populations and low-income settings (6).

Given the interplay of genetic and environmental factors, understanding the familial clustering of RHD is crucial. Family members incorporate socioeconomic and environmental factors, as well as genetic predisposition. Many alleles and genes have been reported to increase susceptibility to RHD and rheumatic fever (7). It has long been proposed that genetic susceptibility plays an important role in the development and progression of RHD (8). However, this observation may be influenced by environmental factors such as overcrowding, poverty, and limited access to healthcare, which are usually shared by family members and are well-known risk factors for RHD.

To assess whether first-degree relatives of patients with ARF or latent RHD have a higher susceptibility to RHD, warranting targeted screening, we conducted a systematic review and meta-analysis of RHD prevalence by echocardiographic screening in first-degree relatives compared to population controls from the same region – geographic area or community.

## Methods

We conducted a systematic review and meta-analysis according to Cochrane recommendations and Preferred Reporting Items for Systematic Reviews and Meta-Analysis (PRISMA) statement guidelines (9,10). This review was prospectively registered with the International Prospective Register of Systematic Reviews (PROSPERO) under the numeric register of CRD42023466262. The methods applied in this research and raw data utilized for this meta-analysis may be shared upon reasonable request to the corresponding author.

### Eligibility criteria and data extraction

The inclusion criteria for this meta-analysis encompassed prospective observational studies that utilized echocardiography to screen first-degree relatives of patients with ARF or latent RHD for assessment of undiagnosed disease and compared with the region population. We chose ARF and latent RHD as index cases because ARF has a high rate of progression to RHD, with approximately 60% of cases developing RHD (11,12). Latent RHD, depending on its severity, also has a high prevalence, ranging from 10% to 50% of the progression rate (13,14). There were no age, sex, or geographic restrictions in the study selection criteria. Excluded from consideration were conference abstracts, retrospective studies, studies that screened households and not family members, and studies without complete confirmatory transthoracic echocardiogram. Our systematic search in September 2023 included four databases—PubMed, Embase, LILACS, and the Cochrane Central Register of Controlled Trials. The search employed a combination of keywords such as *“echocardiography,” “echocardiogram,” “family,” “household,” “relatives,” and “rheumatic.”* The detailed search string can be found in the **Supplementary Appendix**.

Two investigators (AM and GP) independently reviewed findings based on predetermined criteria to assess search results and include studies. Any discrepancies were resolved through consensus or by a senior investigator as a tiebreaker. Two independent authors (AM and GP) extracted data according to specific endpoints and key characteristics of included studies. When data on key outcomes of variables of interest were absent, we contacted authors to request the necessary results for inclusion in our systematic review.

### Endpoints

The primary objective of this study was to determine the prevalence of definite RHD in individuals under 20 years old, as well as overall RHD in those over 20 years old among first-degree relatives of individuals with ARF or latent RHD. Additionally, we conducted a secondary analysis to assess the prevalence of definite RHD alone, noting that the included studies did not apply the new WHF category for subclinical RHD diagnosis (15). Finally, we compared the prevalence of RHD in first-degree relatives to that in the general regional population, excluding individuals with first-degree relatives affected by latent RHD or ARF.

### Quality assessment

Two authors (AM and GP) conducted an independent assessment of the quality of each study using the risk of bias tool developed by Hoy et al. (16). In cases in which differences in assessment arose, consensus was reached through discussion. This evaluation comprises 10 items, each assigned a score of 1 or 0. The cumulative scores contribute to an overall quality assessment categorized as low, moderate, or high risk of bias. A score of 3 or lower indicates a high risk of bias, a score ranging from 4 to 6 suggests a moderate risk and a score from 7 to 10 suggests a low risk of bias. The investigators graded the studies separately, and discrepancies were resolved by consensus before the final grading.

### Statistical analysis

To determine the prevalence of RHD, we employed a single-arm meta-analysis utilizing random-effects generalized linear mixed models (GLMM) and 95% confidence intervals (CI). For secondary analyses involving the direct comparison of incidence rates in specific subgroups, we utilized a Mantel-Haenszel random-effects model to assess the relative risk (RR) between groups, with a corresponding 95% CI. To estimate the between-study variance, we used restricted maximum likelihood.

Statistical significance was determined by p-values less than 0.05. Additionally, we assessed the impact of between-study heterogeneity on the estimates using Cochran’s Q test, the I^2^ statistic, and the Tau^2^ estimate (variance of the effect size parameters across the population of studies). Following Cochrane guidelines, we considered a p-value less than 0.10 in the chi-square test, Tau^2^ >0, or an I^2^ greater than 40% as indicative of substantial heterogeneity, potentially influencing the reliability of the estimates (10).

Moreover, we conducted a leave-one-out sensitivity analysis to identify potential effect modifications in the estimated outcomes. Two authors, AM and AN, independently carried out all statistical analyses using R version 4.3.0 (R Foundation for Statistical Computing, Vienna, Austria) (17).

## Results

### Study selection and baseline characteristics

**Figure 1:**
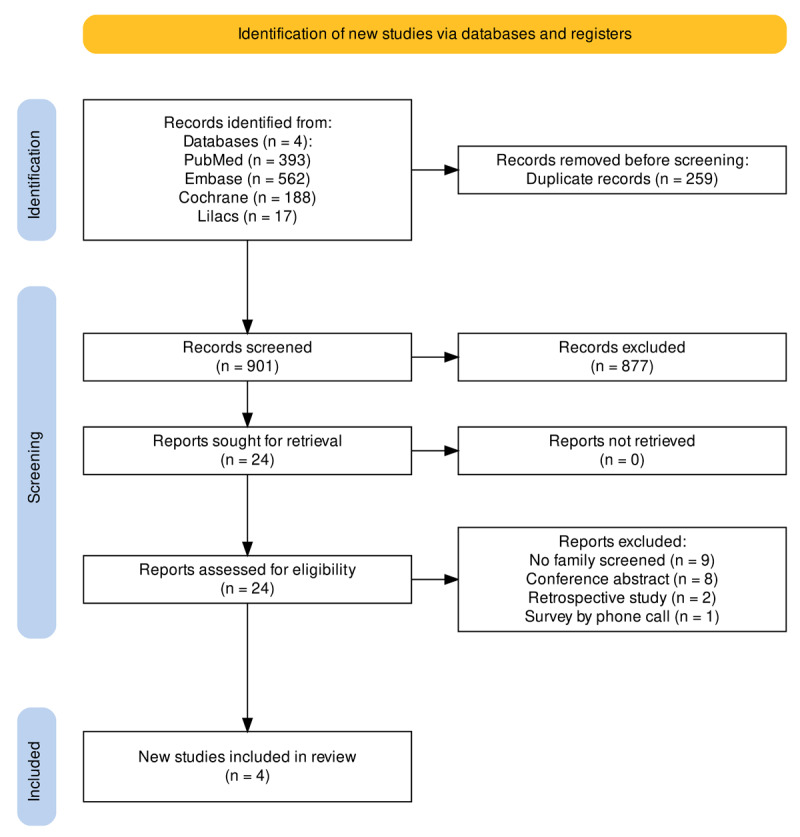
Flow diagram of study selection.

The search across the four databases yielded 1,160 articles and conference abstracts. After removing duplicates, 901 articles remained. Following title and abstract screening, 877 articles were excluded. A full-text review was conducted on 24 articles, and four met our eligibility criteria (Figure 1) (18,19,20,21). The included articles involved the systematic echocardiographic screening of 776 first-degree relatives of patients with a history of ARF/RHD. Among these, 226 (29%) were parents, 415 (53%) were siblings, and 133 (17%) were children of the index case. In three studies, the index cases were diagnosed with latent RHD, while in one study, they were diagnosed with ARF. Additional details on the included studies can be found in Table 1.

**Table 1:** Characteristics of included studies.


STUDY	SCREENING YEARS	COUNTRY	INDEX CASES (N)	FAMILY SAMPLE	PARENTS	SIBLINGS	CHILDREN	POSITIVE CASES	RISK OF BIAS

Aliku, 2016	2015–2015	Uganda	RHD60	235	79	156	–	15	Low

Culliford-Semmens, 2021	2014–2016	New Zealand	ARF70	229	96	133	–	7	Low

Franco, 2022	2020–2021	Brazil	RHD121	226	20	77	129	14	Low

Gemechu, 2021	2017–2021	Ethiopia	RHD28	86	31	49	4	15	Low


In our included studies, Aliku et al. matched participants by age, gender, and school, providing a strong regional match, while Franco et al. included socioeconomic matching within the same city (18,20). Gemechu et al. controlled for age and sex, though a regional match was not specified (19). Culliford-Semmens et al. focused on high-prevalence regions but did not implement explicit matching controls (21). Therefore, we performed a sensitivity analysis with only Aliku et al. and Franco et al. results (18,20).

### Prevalence of RHD in first-degree relatives

**Figure 2:**
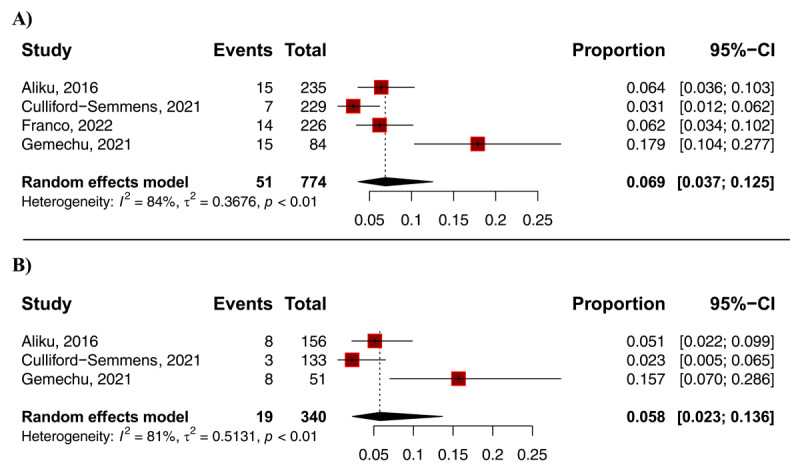
Prevalence of rheumatic heart disease (RHD) in first-degree relatives of index cases; **A –** definite or RHD prevalence (definite for those under 20 years old combined with RHD for those with more than 20 years old); **B-** Definite RHD prevalence (analysis of only first-degree relatives under 20 years old by 2012 WHF diagnostic criteria).

The overall prevalence of RHD or definite RHD in first-degree relatives of index cases was 7.0% (95% CI: 3.7–13%; I^2^ = 84%; Figure 2a). When examining the prevalence of only definite RHD (Stage B or C for the new criteria (15)) was 5.8% (95% CI 2.3–14%; I^2^ = 81%; Figure 2b).

**Figure 3:**
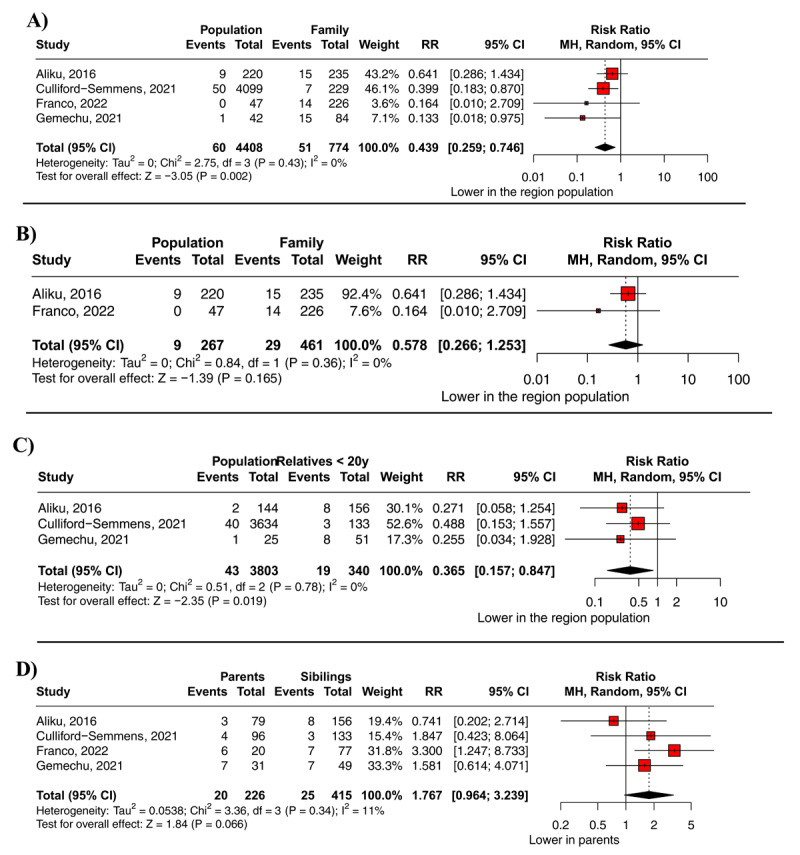
Direct comparison of RHD or definite RHD prevalence; **A**- First-degree relative prevalence *vs*. non-relatives’ prevalence from same Region population; **B**- First-degree relative prevalence *vs*. non-relatives’ prevalence from same Region and same socioeconomic background; **C**- First-degree relative prevalence *vs*. non-relatives’ prevalence from same Region population on definite RHD (region population and family members under 20 years old, the 2012 WHF diagnostic criteria was used); **D**- Parents *vs*. siblings of index cases on the RHD prevalence.

A higher prevalence of RHD or definite RHD was observed in first-degree relatives of index cases compared to the regional sample of non-relatives (RR 0.44; 95% CI 0.26–0.75; p = 0.002; I^2^ = 0%; Figure 3a). The analysis, including only Aliku et al. and Franco et al., did not show statistical relevance for lower incidence in the regional population (RR 0.58; 95% CI 0.27–1.25; p = 0.165; I² = 0%; Figure 3b) (18,20). In a direct comparison between RHD or definite RHD prevalence among siblings and parents of index cases, no significant difference was found (RR 1.8; 95% CI 0.96–3.24; p = 0.066; I^2^ = 11%; Figure 3c). When we compared the family members younger than 20 years old with the region population for the definite RHD, we found a higher incidence in the relative’s group (RR 0.37; 95% CI 0.16–0.85; p = 0.019; I^2^ = 0%; Figure 3d). However, when we compared those older than 20 for RHD, there was no difference (RR 0.57; 95% CI 0.15–2.16; p = 0.404; I^2^ = 53%; **Supplementary Fig 1**).

**Figure 4:**
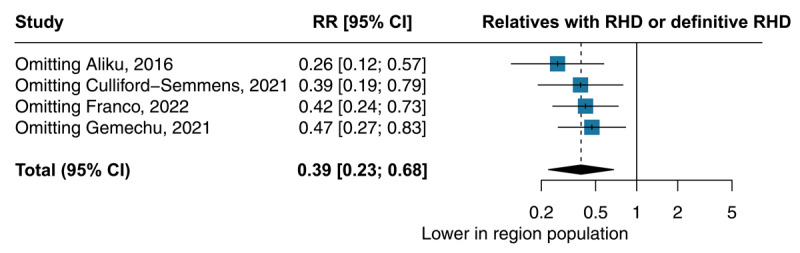
Leave-one-out sensitivity analysis on the direct comparison of RHD: First-degree, relative *vs* Region population.

As a sensitivity analysis, we performed the leave-one-out analysis, which evaluates the influence of each study on the pooled estimated prevalence. The sensitivity analysis for the direct comparison between first-degree relatives and patients without any first-degree relatives with ARF/RHD in the local population showed consistency in our findings and is depicted in Figure 4. Taking the Culliford-Semmens et al. study, the only one with ARF index cases, the leave-one-out analysis resulted in an RR of 0.39 and a CI of 0.19–0.79 (21).

### Quality assessment

All four included studies were deemed to have a low risk of bias. They each directly collected patient data, utilized a first-choice diagnostic method and had robust sample sizes. However, a point was deducted from each study in the fourth criterion due to non-response bias, as not every first-degree relative of index cases participated. The study conducted by Culliford-Semmens et al. additionally lost a point in the ninth criterion (21). This deduction was attributed to the fact that the index cases in this study were children with ARF, rendering the length of the shortest prevalence period for the parameter of interest inappropriate. Further information on the quality assessment is available in **Supplementary Table S3**.

## Discussion

This is the first systematic review and meta-analysis to determine the prevalence of RHD in first-degree relatives of index cases of latent RHD or ARF. We analyzed data from four prospective studies, including 776 first-degree relatives and 279 index cases. The prevalence of RHD in the relative’s group was 7%. In direct comparison, first-degree relatives exhibited a significantly higher RHD or definite RHD prevalence than the region population group. Therefore, first-degree relatives of patients with latent RHD or ARF constitute a high-risk group for RHD.

Echocardiographic-detected RHD has a prevalence of 2.1% in endemic areas and can increase in high-risk groups (6). We found a prevalence of 7% in the first-degree relatives, which is more than three times higher than the prevalence in non-first-degree relatives from endemic areas found in a previous meta-analysis (6). Socioeconomic and environmental factors influence ARF and RHD. Therefore, family members who share these risk factors in their household are likely to be at a higher risk for these conditions (22,23) However, in a series that screened relatives and non-relatives living in the same household, as in the study by Franco et al., relatives seem to have an increased risk, independently of socioeconomic status (20). In addition to these factors, previous studies have shown that family members may share genetic traits associated with an increased risk of developing RHD (20,24,25).

A prior systematic review and meta-analysis compared ARF prevalence in monozygotic versus dizygotic twins, highlighting a significant genetic predisposition to the disease (24). This analysis of over 400 twins revealed a concordance risk of 44% in monozygotic twins and 12% in dizygotic twins, with a heritability estimate of 60% (24). Our findings provide additional evidence of the importance of genetic predisposition in RHD development, alongside the well-established socioeconomic risk factors, such as household crowding.

We initially hypothesized that siblings would have a greater prevalence of RHD than parents due to shared genetic predisposition and similar environmental risk factors during childhood. However, we found no significant differences in RHD between siblings and parents. This may be influenced by three main factors: (i) delay in disease development; (ii) improvements in healthcare and disease control; and (iii) the relatively small sample size of parents and siblings, which leads to high heterogeneity and wider confidence intervals. Since RHD typically develops over time following an ARF attack, siblings may not have had their first ARF or second attack, and parents may have been exposed to ARF for a longer period, increasing their risk of developing RHD (26). Moreover, enhanced healthcare delivery over time, especially access to antibiotic prophylaxis and clinical follow-up, may have recently prevented occurrences of ARF, as demonstrated by the decreased incidence over time in most regions, especially those with better sociodemographic status (27). Thus, comparing rates between siblings and parents may not be reliable, considering the changes in the natural course of the disease over the last few years and the limitation of the meta-analysis due to sample size.

Genetic studies provide valuable insights into disease mechanisms and potential treatments. For example, the GWAS study by Lesley-Ann Gray et al. identified risk HLA haplotypes that strongly bind to streptococcal epitopes, triggering cross-reactive immune responses, which later develop into ARF and RHD (28). This study also highlighted the potential for vaccination targeting non-cross-reactive epitopes. Our study supports the genetic predisposition theory, emphasizing the need for further research to define risk and protective haplotypes better, paving the way for new diagnostic and therapeutic approaches.

Understanding which groups are most likely to develop RHD is crucial for early diagnosis and intervention. Early diagnosis of latent RHD in family members is critical not only for patient information and for exposing the actual burden of the disease to the populations at risk but also for clinical management and research. Despite the lack of a specific therapy targeting the valvular rheumatic alterations, regular administration of penicillin injections every four weeks to children and adolescents with latent RHD can prevent disease progression (5). A sizeable ongoing trial is being conducted to investigate the effects of oral penicillin in this scenario (29). Furthermore, secondary prophylaxis with penicillin is also indicated in adults depending on the severity of the valvular disease, even in asymptomatic patients for long periods (4,30). Actively diagnosing RHD through identifying high-risk groups is crucial for facilitating close follow-up to assess disease progression. Additionally, it is of significant importance in managing cases of atrial fibrillation with valvular etiology, among other advanced complications. As an example of disease management, in patients without RHD, the current recommendations support the use of factor Xa inhibitors. Nonetheless, the INVICTUS trial demonstrated that these drugs increase the risk of mortality and stroke compared to vitamin K antagonists in RHD, highlighting the need for further investigations (31).

Moreover, diagnosing patients with RHD at different stages of valvular damage will open avenues for novel research studies encompassing the entire spectrum of RHD severity and assessing biological, genetic, and socioeconomic predisposing factors. The high prevalence of RHD among family members of index cases suggests a more susceptible group. This group could not only receive better individual care, being candidates for targeted screening and follow-up strategies, but their identification also plays a pivotal role in enabling novel scientific discoveries in RHD. Therefore, more studies are needed better to understand the potential impact of first-relative screening in RHD.

Our study has limitations. First, a limited number of studies were available for inclusion in our analysis, resulting in considerable uncertainty in some estimates Second, the studies differed regarding the index case, which was either ARF or latent RHD. Our findings remained consistent throughout the leave-one-out sensitivity analysis, even when excluding the study with the ARF index case. The Gemechu et al. study reported a higher prevalence of latent RHD among family members (19), which may raise suspicion. However, when we directly compared family members with the regional population, this difference disappeared, indicating a high regional prevalence. Furthermore, the included studies also showed heterogeneity in the quality of the control groups (only two studies had a socioeconomic control group). The analysis of these two studies alone did not achieve statistical significance, highlighting the need for more studies designed with socioeconomic, age, and sex matching. Last, we did not have access to individual patient data, which could have provided a deeper understanding of factors influencing RHD prevalence, including differences between siblings (boys and girls) and parents (mothers and fathers).

## Conclusion

The overall prevalence of RHD in first-degree relatives was 7%, higher than in non-relatives from the same region, indicating that family members of patients with latent RHD or ARF are at greater risk of developing RHD. Further studies are needed to investigate the role of genetic predisposition in RHD, considering the influence of socioeconomic risk factors.

## Data Accessibility Statement

Data are available within reasonable request.

## Additional File

The additional file for this article can be found as follows:

10.5334/gh.1417.s1 Supplementary Files. Supplementary Appendix.

## Abbreviations

RHD: Rheumatic heart disease

ARF: Acute rheumatic fever

PRISMA: Preferred Reporting Items for Systematic Review and Meta-Analysis

PROSPERO: International Prospective Register of Systematic Review

CI: Confidence interval

RR: Risk ratio
