# Supplementary material for: Prevalence of Rheumatic Heart Disease in First-Degree Relatives of Index-Cases: A Systematic Review and Meta-Analysis
Source: Glob Heart. 2025 Mar 10;20(1):24. doi: 10.5334/gh.1417 (PMC11908434; doi:10.5334/gh.1417)
Supplement: Supplementary Files. — Supplementary Appendix. [file gh-20-1-1417-s1.pdf]

Supplementary Appendix

TABLE OF CONTENTS:

Table 1: Search strategies

Table 2: Excluded studies

Table 3: Risk of Bias of included studies

Figure 1: Comparison between relative > 20y with the region population for the RHD incidence.

Figure 2: Leave-one-out for the Definite RHD analysis

References

Table 1: Search strategy

Data base	Search strategy
**EMBASE**	(Echo OR echocardiography OR echocardiographic OR Echocardiogram) AND (Familial OR Family OR Household OR relatives OR Genetic OR community) AND Rheumatic
**PubMed**	(Echo OR echocardiography OR echocardiographic OR Echocardiogram) AND (Familial OR Family OR Household OR relatives OR Genetic OR community) AND Rheumatic
**Cochrane**	(Echo OR echocardiography OR echocardiographic OR Echocardiogram) AND (Familial OR Family OR Household OR relatives OR Genetic OR community) AND Rheumatic
**Lilacs**	(Echo OR echocardiography OR echocardiographic OR Echocardiogram) AND (Familial OR Family OR Household OR relatives OR Genetic OR community) AND Rheumatic

Table 2: Full text reading excluded studies.

Study	Exclusion criteria
Franco, 2021[1]	Conference abstract
Nasciemento, 2021[2]	
Ghamrawy,2019[3]	
Culliford-Semmens, 2018[4]	
Webb, 2017[5]	
Webb, 2016[6]	
Aliku, 2015[7]	
Huang, 2015[8]	
Francis, 2019[9]	No first-degree relative screening
Scheel, 2019[10]	
Ba-Saddik, 2011[11]	
Rizvi, 2004[12]	
Veasy, 1987[13]	
Sriharibabu, 2013[14]	
Abdel-Moula, 1998[15]	
Paar, 2010[16]	
Sptzer, 2015[17]	
Ravisha, 2003[18]	Retrospective study
Vinker, 2010[19]	
Perelini, 2015[20]	Survey by phone call

Table 3: Risk of Bias by Hoy et al. [21]

Study	Criteria 1	Criteria 2	Criteria 3	Criteria 4	Criteria 5	Criteria 6	Criteria 7	Criteria 8	Criteria 9	Criteria 10	Risk of Bias
**Aliku, 2016**	1	1	1	0	1	1	1	1	1	1	Low
**Culliford-Semmens, 2021**	1	1	1	0	1	1	1	1	0	1	Low
**Franco, 2022**	1	1	1	0	1	1	1	1	1	1	Low
**Gemechu, 2021**	1	1	1	0	1	1	1	1	1	1	Low

Figure 1: Comparison between relative > 20y with the region population for the RHD incidence.

Study	Population Events	Population Total	Relatives > 20y Events	Relatives > 20y Total	Weight	RR	95% CI
Aliku, 2016	7	76	3	79	33.0%	2.425	[0.651; 9.037]
Culliford–Semmens, 2021	10	465	4	96	35.9%	0.516	[0.165; 1.611]
Franco, 2022	0	46	13	185	15.5%	0.148	[0.009; 2.440]
Gemechu, 2021	0	17	7	31	15.5%	0.120	[0.007; 1.978]
**Total (95% CI)**	**17**	**604**	**27**	**391**	**100.0%**	**0.565**	**[0.148; 2.159]**

Heterogeneity: $Tau^2 = 0.9668$; $Chi^2 = 6.40$, df = 3 (P = 0.09); $I^2 = 53\%$
Test for overall effect: Z = −0.84 (P = 0.404)

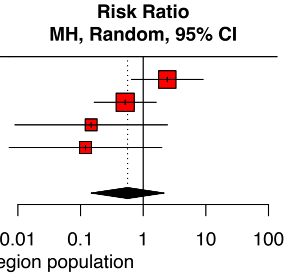

Figure 2: Leave-one-out for the Definite RHD analysis

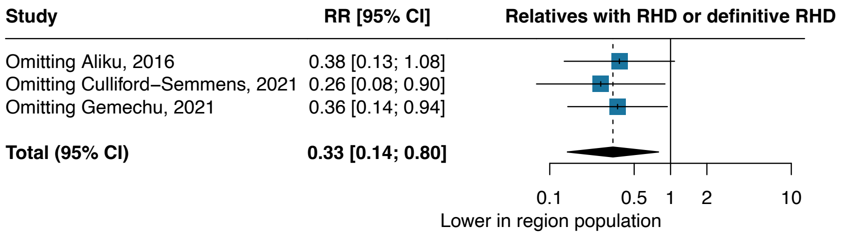

Reference:

1. Franco J, Nascimento BR, Oliveira K, Barbosa M, Arantes NF, Beaton AZ, et al. Evaluation of family risk of rheumatic heart disease with systematic echocardiographic screening: data from the provar+ study. Circulation [Internet]. 2021;144(SUPPL 1). Available from: https://www.embase.com/search/results?subaction=viewrecord&id=L636897239&from=export

2. Nascimento BR, Franco J, Beaton A, Oliveira KKB, Barbosa M, Ribeiro A, et al. ECHOCARDIOGRAPHIC SCREENING FOR EVALUATION OF FAMILY RISK OF RHEUMATIC HEART DISEASE - DATA FROM THE PROVAR+ STUDY. J Am Coll Cardiol. 2021;77(18):1712.

3. El Ghamrawy AEDM, Abd El-Wahab EW, Nabil NA. Trends in rheumatic heart disease in Egypt (2006-2018): Data from the national rheumatic heart prevention and control program. Eur Heart J. 2019;40((El Ghamrawy A.E.D.M.) El Mahalla Cardiac Center, Almahalla Alkobra, Egypt):1696.

4. Culliford-Semmens N, Tilton E, Wilson N, Stirling J, Doughty R, Gentles T, et al. The New Zealand familial echo study reveals high prevalence of rheumatic heart disease amongst parents and siblings of children with rheumatic fever: Echocardiography should be offered to first degree relatives. J Paediatr Child Health. 2018;54((Culliford-Semmens N.; Tilton E.; Wilson N.; Stirling J.; Gentles T.; Lennon D.; Webb R.) Starship Children’s Hospital, Auckland, New Zealand):6.

5. Webb R, Culliford-semmens N, Tilton E, Nicholson R, Doughty R, Lennon D, et al. The New Zealand familial echo study reveals high prevalence of rheumatic heart disease amongst parents and siblings of children with rheumatic fever echocardiography should be offered to first degree relatives. Cardiol Young. 2017;27(4):S483.

6. Webb R, Culliford-Semmens N, Mow AC, Doughty R, Tilton E, Peat B, et al. Prevalence of rheumatic heart disease and other echocardiographic abnormalities in polynesian young adults in South Auckland, New Zealand. Glo Heart. 2016;11(2):e63.

7. Aliku T, Beaton A, Scheel A, Tompsett A, Lwabi P, Sable C. Evaluating the risk of rheumatic heart disease among family members of children identified with latent RHD in school-based echocardiographic screening. Circulation [Internet]. 2015;132((Aliku T.) Cardiology, Gulu Univ, Gulu, Uganda). Available from: https://www.embase.com/search/results?subaction=viewrecord&id=L72181263&from=export

8. Huang JH, Favazza M, Legg A, Holmes K, Armsby LR, Pilgrim T, et al. Echocardiographic screening for rheumatic heart disease in children in American Samoa. J Am Soc Echocardiogr. 2015;28(6):B97.

9. Francis JR, Gargan C, Remenyi B, Ralph AP, Draper A, Holt D, et al. A cluster of acute rheumatic fever cases among Aboriginal Australians in a remote community with high baseline incidence. Aust N Z J Public Health. 2019 Jun;43(3):288–93.

10. Scheel A, Ssinabulya I, Aliku T, Bradley-Hewitt T, Clauss A, Clauss S, et al. Community study to uncover the full spectrum of rheumatic heart disease in Uganda.

Heart. 2019 Jan;105(1):60–6.

11. Ba-Saddik IA, Munibari AA, Al-Naqeeb MS, Parry CM, Hart CA, Cuevas LE, et al. Prevalence of rheumatic heart disease among school-children in Aden, Yemen. Ann Trop Paediatr. 2011;31(1):37–46.

12. Rizvi SF, Khan MA, Kundi A, Marsh DR, Samad A, Pasha O. Status of rheumatic heart disease in rural Pakistan. Heart. 2004 Apr;90(4):394–9.

13. Veasy LG, Wiedmeier SE, Orsmond GS, Ruttenberg HD, Boucek MM, Roth SJ, et al. Resurgence of acute rheumatic fever in the intermountain area of the United States. N Engl J Med. 1987 Feb 19;316(8):421–7.

14. Sriharibabu M, Himabindu Y, Kabir Z. Rheumatic heart disease in rural south India: A clinico-observational study. J Cardiovasc Dis Res. 2013 Mar;4(1):25–9.

15. Abdel-Moula AM, Sherif AA, Sallam SA, Mandil AM, Kassem AS, Zaher SR. Prevalence of rheumatic heart disease among school children in Alexandria, Egypt: a prospective epidemiological study. J Egypt Public Health Assoc. 1998;73(3–4):233–54.

16. Paar JA, Berrios NM, Rose JD, Cáceres M, Peña R, Pérez W, et al. Prevalence of rheumatic heart disease in children and young adults in Nicaragua. Am J Cardiol. 2010 Jun 15;105(12):1809–14.

17. Spitzer E, Mercado J, Islas F, Rothenbühler M, Kurmann R, Zürcher F, et al. Screening for Rheumatic Heart Disease among Peruvian Children: A Two-Stage Sampling Observational Study. PLoS One. 2015;10(7):e0133004.

18. Ravisha MS, Tullu MS, Kamat JR. Rheumatic fever and rheumatic heart disease: clinical profile of 550 cases in India. Arch Med Res. 2003;34(5):382–7.

19. Vinker S, Zohar E, Hoffman R, Elhayany A. Incidence and clinical manifestations of rheumatic fever: a 6 year community-based survey. Isr Med Assoc J. 2010 Feb;12(2):78–81.

20. Perelini F, Blair N, Wilson N, Farrell A, Aitken A. Family acceptability of school-based echocardiographic screening for rheumatic heart disease in a high-risk population in New Zealand. J Paediatr Child Health. 2015 Jul;51(7):682–8.

21. Hoy D, Brooks P, Woolf A, Blyth F, March L, Bain C, et al. Assessing risk of bias in prevalence studies: modification of an existing tool and evidence of interrater agreement. J Clin Epidemiol. 2012 Sep;65(9):934–9.
